# The Hebrew Wards at the London Hospital

**Published:** 1890-06-21

**Authors:** 


### The Hospital Workshop.

(By our Special Commissioner.)

### No. XXVII.—THE HEBREW WARDS AT THE LONDON HOSPITAL.

A large Hebrew population resides in our metropolis. Although the possessors of enormous wealth yet at the same time many thousands of their number pass their lives in abject poverty. The latter class is largely made up of immigrants from foreign countries. When one considers the wealth, the charity, the deeply-rooted customs, and the strong national feelings that characterise the race, it seems strange that so small a provision exists for special medical treatment of the poorer members of their community. There is a small hospital for Spanish and Portuguese Jews in the Whitechapel Road, and many go to the German and Metropolitan Free Hospitals. An attempt has also been recently made at a newly-founded institution to provide special wards. The only place, however, where such accommodation has been systematically afforded is the London Hospital in the East-end. Two wards were opened there in 1840, the male ward, containing fifteen beds, dedicated to the memory of its founder, Abraham Goldsmid; and the female, with twelve beds, to Baron N. Meyer de Rothschild. During the years 1868 to 1888 it appears that 8,164 patients were admitted. That this separate accommodation is quite inadequate to meet the wants of the Jewish colony need hardly be pointed out. The sick, however, are not allowed to suffer in consequence, for every patient who really ought to come in is admitted to the general wards and supplied with food from the Hebrew kitchen. As vacancies occur these outlying patients are transferred to the special beds. In the kitchen alluded to all the customs in relation to the use of vessels and the preparation of food are carried out under a Jewish cook.

In the male ward is a Polish boy of ten who tells us his name is Abraham; that he has had a bad leg (hip joint disease) since something that sounds like "Pisah," and which the sister-in-charge interprets as "Passover." His father is a tailor by trade. On the other side of the ward is a young man of twenty, propped up in bed, white and gasping, evidently in a very critical condition. Another patient is giving him a bit of ice to suck from time to time. With some difficulty he tells us that he worked as a hatter at Greenwich until nine months ago, when he came here. He was born in Whitechapel, and his parents, who are Poles, work at tailoring. The poor fellow is in an advanced stage of dropsy. He is extremely ill, and his friends are accordingly permitted to see him at any time. Under ordinary circumstances Friday afternoon is the stated visiting day. Two visitors are allowed to each patient. They bring with them butter and sugar, which are not provided by the hospital. One woman was carrying a pillow for her son. The inmates of these wards are allowed to have their own feather pillows, a privilege not accorded to the general patient.

A large number of the sick hail from the tailors' workshops, the "sweating dens" about which so much has been heard of late years. Many more are bootmakers, or follow the trades of feather-cleaning, cigar-making, millinery, mantle-work, "machining," or the manufacture of picture-frames. Your commissioner was informed on these points by a handsome visitor with dark eyes and olive skin, who was herself employed in button-hole making. According to her statement, it is the daughters, not the wives, who usually do the work. There are also a good many furriers. The traders look out for foreigners directly they come off the ships, and generally contrive to bind them as apprentices for a certain number of years.

A little girl of ten is sitting by the fire in the female ward. Her wan white face makes a sharp contrast with her raven hair. The crutches and the high boot tell the tale of chronic hip joint disease. She has not yet quite learnt to get about with her "Thomas's splint," but a little time will soon set that right. The poor child has been an invalid for years, and has been an inmate of the Children's Hospital in Great Ormond Street, and also of the German Hospital. It is raining outside, a matter of no little anxiety, for her mother is going to take her back home to-day "in a tram."

It has often been remarked that the London Hospital is a household word among the inhabitants of the East-end, most of whom have either themselves been under treatment, or have had friends, relatives, or dependents there. One patient gave a history strongly illustrating this point. Twenty-three years ago she was under treatment in this ward for "a kind of stroke" which caused loss of power of the right arm during a period of five weeks. Since that time she has married and had a family of fourteen children. Of these the following have been in the hospital. 1. *Matilda B.*, for nævus ("birth-mark") of the forehead, head and back, admitted thrice and finally cured. 2. *Angel B.*, admitted for eleven weeks, after severe scald from a kettle of boiling water. 3. *Barnet B.*, two joints of thumb of right hand amputated after injury from cog wheel. He is now able to work well at "rivetting" in his trade, which is the "boot line." 4. *Joey B.*, diseased shoulder joint, following a blow, lasted for four years. Was in for six months on one occasion, and three months on another. Underwent an operation and recovered, can now "use a good sledge-hammer with that arm." Also in boot line.

There are two main Jewish colonies in the East-end. One is in Spitalfields, the other at the back of Church Lane in the Minories. There are whole streets full of Dutch colonists near Spitalfields Church, from which fact the district was formerly known as "Little Holland." Many of the names over the shops in the Whitechapel Road bear evidence of Hebrew origin. Samuels, and Aarons, and Levys abound, but there are many rarer ones of foreign extraction, such as Catmur, Tobin, Crona, Bonarius, Rutzen, Apfel, Montschein, and Hemus.

In the wards described Hebrew customs are respected and carried out without hindrance. Over the door, for instance, is a large round cake of "Kosher," or Passover bread, left from the feast of one year until the next, and behind the door-post is fastened a piece of parchment on which the Commandments are inscribed. Among matters of ceremonial a "watcher" attends the dying with candles, and a number of friends gather round the bed and raise the death lamentation.

The fact that the habits and customs of this ancient race are thus respected is a sign of the progressive spirit of modern charity. The Gospel of good-will to all men could hardly be embodied with greater force than that which is set forth in the Hebrew wards at the London Hospital.

